# Genotyping and Phylogenetic Analysis of Group B Streptococcus by Multiple Locus Variable Number Tandem Repeat Analysis in Iran

**DOI:** 10.22086/gmj.v0i0.1121

**Published:** 2018-03-28

**Authors:** Farzaneh Khodaei, Behrooz Sadeghi Kalani, Naser Alizadeh, Alka Hassani, Mohammad Najafi, Enayatollah Kalantar, Abbas Amini, Mohammad Aghazadeh

**Affiliations:** ^1^Immunology Research Center, Department of Medical Microbiology, Faculty of Medicine, Tabriz University of Medical Sciences, Tabriz, Iran; ^2^Department of Medical Microbiology, Iran University of Medical Sciences, Tehran, Iran; ^3^Department of Medical Microbiology, Tabriz University of Medical Sciences, Tabriz, Iran; ^4^Department of Biochemistry, Iran University of Medical Sciences, Tehran, Iran; ^5^Department of Microbiology and Immunology, School of Medicine, Alborz University of Medical Sciences, Karaj, Iran; ^6^School of Computing, Engineering and Mathematics, University of Western Sydney, Kings wood, NSW 2751, Australia

**Keywords:** Antimicrobial Susceptibility, Capsular Antigen, Group B *Streptococcus*, MLVA, Typing, *Pilus* Island

## Abstract

**Background:**

Group B streptococcus (GBS), also known as Streptococcus agalactiae, is well known as a causative agent for neonatal invasive diseases; it is also a major pathogen in adults. Analytic epidemiology is required to monitor the clinical isolates of GBS. However, there is insufficient information on the genetic background of GBS in Iran, and this information is needed to guide and develop a GBS vaccine.

**Materials and Methods:**

In total, 90 well-char - acterized GBS isolates were collected from April 2014 to August 2015. In this study, molecular typing was used to disclose a relationship between the multiple-locus variable number tandem repeat analysis (MLVA) types, serotyping, and pilus islands. The isolates were characterized by the types of capsular polysaccharides and pilus islands and were examined by MLVA to study the epidemiological relationship of isolates.

**Results:**

The results indicate that there is a significant relationship between the distribution of serotypes and pilus island genes; GBS isolates were differentiated into 12 types by capsular polysaccharides and pilus islands analysis. The discriminatory power of an MLVA analysis was high based on the five most variable numbers of tandem repeat loci and 44 MLVA types that were identified.

**Conclusion:**

This study has provided useful insights into the genetic heterogeneity of GBS isolates in Tehran and Alborz, Iran. The extensive distribution of pilus islands in various serotypes and MLVA types throughout the GBS population refers to the advancement of the pilus-based GBS vaccines.

## Introduction

*Streptococcus agalactiae*,or group B streptococcus (GBS),may be one of the common causative agents of neonatal diseases such as respiratory diseases, septicemia, and meningitis in the first 6 days of life (early-onset disease), as well as from 1 week up to 3 months old (late-onset disease) [1]. Recently, GBS has been increasingly associated with invasive disease in no pregnant adults and immunocompromised patients [2]. GBS isolates were initially differentiated based on the variations in capsular polysaccharides (Ia, Ib, and II to IX) [3]; for example, serotype III causes a considerable proportion of infant infections [4]. Serotyping is one way to differentiate isolates, but this method does not have the enough differentiating power to discriminate between isolates [5]. The analysis of bacterial isolates using multiple genotyping methods provides significant information for creating a genetic relationship between isolates for epidemiological purposes and phylogenetic research [6]. Among these techniques, pulsed-field gel electrophoresis (PFGE) has more discriminatory power. However, the PFGE technique is required as a standardized protocol for reproducible results. A standardized Pulse Net PFGE protocol is functional; however, it might be difficult to have exact inter laboratory results [7]. Among the next generation of molecular subtyping techniques, multiple-locus variable number tandem repeat analysis (MLVA) is an efficient typing method; its fast and resolving power is utilized to create the DNA fingerprinting of a bacterial isolate through polymerase chain reaction (PCR) amplification for epidemiological studies [5,8]. The MLVA assay is a particular identification method that distinguishes a variable number of tandem repeat (VNTR) loci. VNTRs are short-to-long nucleotide sequences (20-100 bp) that differ in the number of copies in bacterial genomes. They assess the number of repeats in a set of VNTR loci dispersed throughout the bacterial genome. They have been extensively used for genotyping isolates of various bacterial species [5]. MLVA is also useful in biological research, forensic science, and clinical genetics. Several studies in Iran[9,10] have reported the prevalence of GBS serotypes and changes and increased antibiotic resistance for GBS. However, this information is not well understood due to limited comprehensive studies. The aim of this study is to determine the genetic diversity and phylogenetic analysis of GBS isolates through the MLVA method, and with the help of an epidemiological study, their association with different serotypes, antibiotic resistance patterns, and pilus islands genes in clinical isolates of GBS in an epidemiological study.

## Materials and Methods

### Study Design

This study is a cross-sectional study. From April 2014 to August 2015, 14,686 samples were evaluated in terms of the presence of GBS from two clinical laboratories of Tehran and Alborz, Iran. A total of 90 positive isolates were used in invasive and noninvasive isolates among adults. Among 90 isolates, four totally different sets of isolates were examined: 13 (14.5%) invasive isolates, with the origin being blood; 13 (14.5%) noninvasive isolates, 4 (4.4%) spermatic fluids, 26 (28.8%) colonizing isolates collected from vaginal secretions of pregnant women, and 34 (37.7%) urine samples from healthy patients. All GBS isolates were identified based on biomedical standard protocols [11]. The identification of GBS was accomplished using a multiplex PCR method to identify the nine capsular polysaccharide GBS serotypes [12]. Susceptibility testing for erythromycin and clindamycin was performed by disk diffusion and D-zone test [13].

### DNA Extraction

GBS isolates were incubated overnight at 37°C on a Tryptic soy agar with 5% sheep blood (*Pronadisa*, Madrid, Spain). A total DNA template of all GBS isolates was extracted with the genomic extraction kit (Tissue & Cell Genome DNA Purification Kit [Gene Mark], Taiwan) following the manufacturer’s instructions. The quality of the genomic DNA was examined by gel electrophoresis, and the quantity and purity were measured with the NanoDrop 1000 apparatus (NanoDrop TM One C, Thermo Fisher Scientific).

### Identification and Selection of Variable Number of Tandem Repeat Loci, Primer Design 

With the help of the Tandem Repeats Finder (TRF) Software 4.09, more than 100 potential tandem repeats (TRs) were found in the two fully sequenced GBS strains: 2603 V/R (GenBank accession no. AE009948.1) and A909 (GenBank accession no. CP000114.1), using the Tandem Repeats Finder (TRF) software 4.09. After the identification of all TRs common to each strain, five TRs were selected between these two strains, which were of a completely different variety [14] as shown in Table-1. The calculated amplicon size, repeat length, number of repetitions, and the deduced size of flanking regions were examined. The loci were consecutively termed TR1 through TR5, and the allele number was associated with the number of repeats. Also, a null allele (0) was chosen when no amplicon was identified. MLVA of the genetic GBS was conducted to identify the relationship between the GBS isolates and polymorphisms in five VNTR loci. Primers were designed using primer 3 plus software, which was available at http://www.bioinformatics.nl/primer3plus; therefore, this was done to amplify the targets in all GBS isolates whose loci were present. Five loci were recommended for the subsequent MLVA as shown in Table-2. In this study, all primers except primer locus 5 were designed [7]. The analysis of TRs was usually performed with singleplex PCRs in 25 μl of the reaction mixture containing 10 ng DNA template, 200 μM of each dNTP, 1 × PCR Buffer, 2 mM MgCl_2_, 1 unit of *Taq*DNA polymerase, and forward and reverse primers (0.5 μM). Cycling was performed on the Eppendorf 5331 MasterCycler Gradient under the following conditions: initial denaturation at 95°Ϲ for 5 min, followed by 31 cycles of denaturation at 94°Ϲ for 1 min, with an annealing temperature of 58ºC and annealing time of 60 Sec, elongated at 72°Ϲ for 1 min. A final extension step was performed at 72°Ϲ for 8 min. The PCR products were analyzed on 2% agarose gel electrophoresis.

**Table-1:** Properties of 5 Assumed Variable Genetic Loci in *Streptococcus Agalactiae*

**No**	**Ref Strain**	**Period** **Size**	**Min-max size of amplicons (bp)**	**Copy** **Number**	**No. of alleles**	**K-value** ^4^	**Consensus size**	**Putative function**	**Hunter-Gaston diversity index**		**Simpson’s diversity index**	
									**CI**	**HGDI**	**CL**	**SDI**
**TR1**	2603 V/R	18	132-546	3.8	4	11	18	Hypothetical protein	0.857-0.900	0.878	0.847-0.890	0.869
**TR2**	A909	48	281-1529	13.2	10	4	48	FbsA	0.560-0.670	0.615	0.553-0.663	0.608
**TR3**	A909	159	272-1226	4.7	3	8	159	Hypothetical protein	0.760-0.841	0.800	0.751-0.832	0.791
**TR4**	A909	12	253-1000	22.6	7	6	12	Surface adhesion protein	0.999-1.000	0.683	0.619-0.732	0.676
**TR5**	A909	18	139-553	5.8	6	91	18	Hypothetical protein	0.999-1.000	1.000	0.949-0.950	0.989

1. SID: Simpson's diversity index 2. CI: Confidence Intervals 3. HGDI: Hunter-Gaston diversity index 4. K-value: Number of different repeats present at each locus in this sample set

**Table-2:** Sequence of the Primers Used in This Study

**No**	**Forward Sequence (5'->3')**	**Reverse Sequence (5'->3')**	**Coordinate**
**TR_1_**	TCATTATGTAAATGGTGGTGTTGA	TGGGTTTTATGTCCCTCTTCA	666580-666513
**TR_2_**	TGACTGTTTGTTAGAGTCACCTTGA	TTTGGCTTTATATGGGAGTGC	1118269-1118901
**TR_3_**	TTTGAAAAGTGTAACACTAGCTCCA	GGAGCATTCGTAGCTCTTGG	1745897-1746642
**TR_4_**	TTTTTAACCGCCAAGTTTCC	CCACTGATCAAGCAAATCAA	1993522-1993792
**TR_5_**	GTTGATAAAGTTGATGTTCCG	AGCCTTCTTCAACTATAGGTG	746015--746118

### Data Analysis

The repeat units of each locus were calculated by subtracting the size of flanking regions (offset size) from PCR amplicon size and then dividing the distinction by the repeat size. Clustering of the MLVA profile was performed using BioNumerics software, version 6.0 (Applied Maths, Ghent, and East Flanders, Belgium). Each distinctive allelic string was classified as a distinctive MLVA type (MT). A dendrogram was made with the help of unweighted pair group method with arithmetic mean (UPGMA) clustering based on the categorical coefficient. The identical weight was given to dissimilar large and small numbers within the repeats at every locus. Simpson’s index of diversity, Hunter-Gaston diversity index, and 95% confidence intervals (CI) were calculated, which was available at http://www.hpa-bioinfotools.org.uk/cgi-bin/DICI/DICI.pl; as shown in Table-1 [15].

### Ethics Statements

The study protocol was approved by the medical research and Ethics Committee of the Tabriz University of Medical Science (IR.TBZMED.REC.1395.439).

## Results

**Figure-1:**
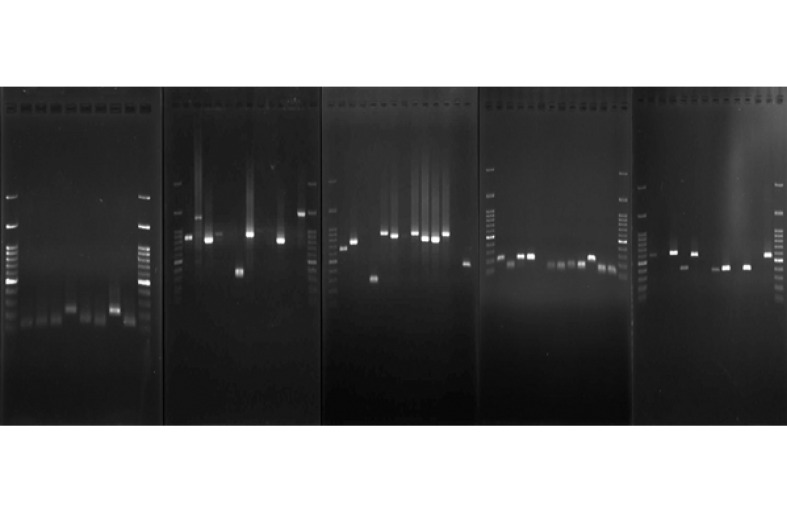
The polymorphism of five VNTRs is shown by agarose gel electrophoresis of PCR products.

**Figure-2:**
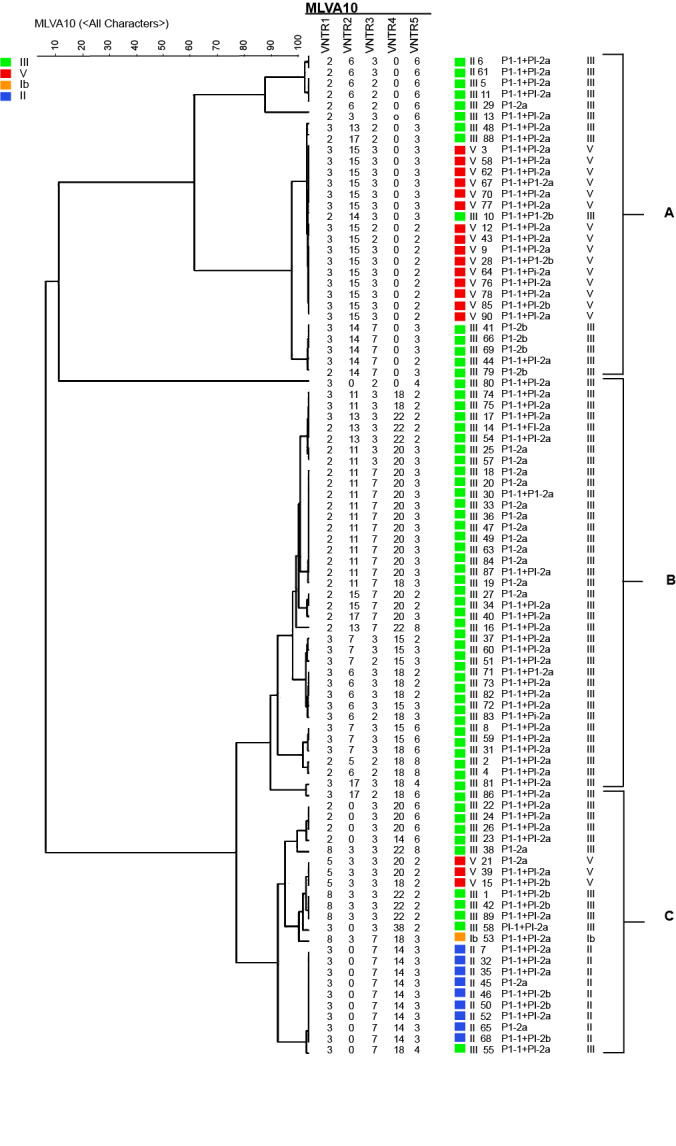
Cluster analysis of 90 *S. agalactiae* isolates using the UPGMA (unweighted-pair group method using average linkages) algorithm, generated with Bionumerics 6.0 software. Clusters are classified on the basis of the level of genetic relatedness, serotypes and pilus island genes. This dendrogram consist of two major clusters designated as A and B and one smaller cluster is known as C.

In this study, 90 GBS were selected out of 14,686 samples' positive cultures. Additionally, the five VNTRs were amplified from all 90 isolates. MLVA was done by individual PCRs and agarose gel electrophoresis of the amplicons as shown in (Figure-1). A phylogenetic tree was made using MLVA profiles; in total, this dendrogram has three clusters that consist of two major clusters designated as A and B and one smaller cluster known as C, as shown in (Figure-2). A diversity index is a quantitative measure that indicates that there are several different types (such as species) in a dataset, and is actually used as a measure of the discriminatory power [1], Calculated values for Simpson’s and Hunter-Gaston diversity index indices demonstrated that TR2 had the lowest diversity (D=0.608), whereas TR5 was the most diverse (D = 0.989). The combination of five loci with the high and low degrees of variability was adequate for molecular subtyping of GBS isolates.

The copy numbers of each VNTR locus are listed in Table-1. The 90 *S. agalactiae* isolates, based on the unique MLVA profiles, were discriminated into 44 different MTs, and a total of 27 MLVA genotypes were shown by one isolate. Among the five loci examined, TR2, TR4, and TR5 were more variable compared to TR1 and TR3. Among 44 MLVA types, MT 18, MT 43, MT 8, and MT 6 were the dominant subtypes, accounting for ten, nine, seven, and six isolates, respectively. The 90 clinical isolates belonged to four different serotypes III, V, II, and Ib, accounting for 68.88%, 20%, 10% and 1.11% respectively. The capsular serotype III and V were usually not limited to a specific MT. When the MTs were analyzed with respect to serotype, isolates of serotype V and especially II (MT43) were mostly present in a single MLVA type. In contrast, the genotypes of serotype III isolates were more heterogeneous. Furthermore, the majority of serotype III isolates belonged to cluster B. Differentiation of resistance phenotypes among erythromycin-resistant streptococci was observed in 31 of 90 isolates. A total of 34.4% of the clinical isolates showed antibiotic resistance patterns to erythromycin antibiotics. The resistance to macrolide was not related to a single cluster, MT, and specific serotype. It was distributed unevenly in all clusters. Resistance to erythromycin and clindamycin (cMLS_B_) was distributed among GBS isolates (N=13) classified in several MTs. The inducible macrolide-lincosamide-streptogramin B (iMLS_B_) were seen in two MTs (21, 38), and M phenotypes were grouped in several different MTs such as cMLS_B_ phenotype, as shown in Table-3.

**Table-3:** Presents the Relationships Between Characterization of GBS Using Multiple-Locus Variable-Number Tandem-Repeat Analysis

**Origin of isolates (No. Of isolates)**					**MLVA genotypes**	**Serotypes**	**Erythromycin resistance phenotype**
**Blood** **(N=13)**	**Vaginal carriage** **(N=34)**	**Urinary** **tract infections in women** **(N=13)**	**Spermatic fluid** **(N=4)**	**Pregnant women** **(N=26)**			
		1			2	III	cMLS_B_^1^
	1				4	III	M^3^
				1	5	III	L^4^
	1				6	V	L
1					6	V	M
1					6	III	M
				1	6	V	cMLS_B_
		1			6	V	cMLS_B_
1					8	V	M
		1			8	V	cMLS_B_
	1				10	III	cMLS_B_
	1				13	III	cMLS_B_
				1	14	II	L
	1				15	III	cMLS_B_
	1				16	III	cMLS_B_
	1				16	III	cMLS_B_
	1				16	III	cMLS_B_
1					16	III	cMLS_B_
	1				16	III	L
1					20	III	M
1					21	III	iMLS_B_^2^
				1	26	III	L
1					28	III	L
	1				28	III	M
				1	30	III	M
				1	31	III	M
				1	34	III	M
1					34	III	L
	1				38	V	iMLS_B_
	1				40	III	cMLS_B_
		1			43	II	cMLS_B_

1. cMLS_B_ phenotype: resistance to both erythromycin and clindamycin 2. iMLS_B_ phenotype: resistance to erythromycin, susceptibility to clindamycin (positive D test) 3. M phenotype: resistance to erythromycin, susceptibility to clindamycin (negative D test) 4. L phenotype: susceptibility to erythromycin, resistance to clindamycin

The highest number of isolates (*n* = 69; 76%) included PI-1 plus one of the two PI-2 variants, whereas 21 isolates (23.3%) displayed only one type of PI-2a or PI-2b exclusively. For the MTs showed by more than three isolates, certain relationships were identified between the presence of a particular pilus island profile and the MT status, eight of the isolates ten classified in MT18 only showed PI-2a profiles, while MT 6 displayed exclusively for PI-1 + PI-2a profiles. Seven of the isolates nine classified in MT43 contained PI combination. The presence of PI-2b was only observed in the cluster A and classified into the two MTs, MT10, and MT12, as shown in (Figure-2).

## Discussion

*S. agalactiae* is known to be a frequent colonizing agent in pregnant women and is a major contributor to neonatal meningitis and septicemia. Nevertheless, over the past years, GBS has been increasingly related to invasive disease in nonpregnant adults [2], especially those with underlying illness such as diabetes [3]. In the current study, the researchers determined genetic diversity and phylogenetic analysis of GBS isolates by the MLVA method for the purpose of the epidemiological study. MLVA is a comparatively new methodology of typing with many advantages over other procedures. VNTR markers show a comprehensive variability. Combining VNTR loci with numerous variables, the values of the different variables may be used to detect different levels of genetic dependence between bacterial isolates [4]. This method can also be useful for discriminating the isolates of the same serotype. A higher degree of genetic diversity was displayed among GBS isolates, despite the close association of several GBS isolates, as surveyed by the serotyping, antibiotic resistance pattern and the presence of pilus islands. In the present study, the 90 GBS clinical isolates included invasive and noninvasive isolates among adults, which were discriminated into 44 different MTs, and a total of 27 MLVA genotypes were shown by one isolate. The results obtained in the present study showed high allelic diversity in the number of repeats of tandem repeats (TR2 and TR5) among GBS isolates, which is comparable to the reports by Haguenoer *et al*. at the research project [5]. The results of this study indicate that the identical capsule genotype was shared by different MTs. This finding could also be due to the horizontal transfer of capsular genes [6]. In this study, it was observed that the isolates belonging to the type II capsule were limited to MT1, demonstrating that the genetic structure of this serotype may be well preserved. Previous studies showed that serotyping alone was not markedly sufficient to identify a phylogenetic lineage distinction in a study population [7,8]; as a result, MLVA with serotyping could be a better way to distinguish between the study population. The findings in this study are in agreement with those obtained by Ouaguiri *et al*. [9]. The results of the antibiotic susceptibility assessment indicate an increase in antibiotic resistance among GBS clinical isolates in Iran and elsewhere in the world [10-12]. This emergence and expansion of antibiotic resistance can be due to the dissemination of clones and the horizontal transmission of resistance genes. It has been reported that increased levels of GBS resistance to macrolide have occurred [13]. Resistance to erythromycin has been extended to all serotypes [14,15]; this finding was similar to the result obtained in the present study. Resistance has also been spread in several genetic lineages. Clonal expansion is additionally important in macrolide resistance; Only resistance to macrolide was significantly correlated with serotype V, which was observed in the study of Uh *et al*. [14]. In the current study, for the first time, the association between the presence of PI and the distribution of clones with MTs among Iranian GBS isolates were interpreted. Our results were consistent with the study performed in Italy by Margarit *et al*. [16], and these results demonstrated that a pilus-based vaccine may broaden protection against GBS disease. Studies carried out to reflect the fact that all isolates studied had at least one pilus island, and the most common profile presence of the PI-1 + PI-2a combination has been considered in several lineages throughout the world [17,18]; as well as, it is close presence in isolates belonging to the three clusters of the phylogeny tree and its relation with the maternal colonization. For the MTs shown by more than three isolates, certain relations were noticed between the presence of a particular pilus island profile and the MT. Although 60% of the isolates carried the PI-1 + PI-2a and PI-2a profiles, mainly in those of serotype III in several MT, the PI-2b alone and PI-1 + PI-2b profiles were identified only in 8.8% of the isolates, principally in those of serotype III and MT 10 and MT 40. Both *cps* serotypes and pilus proteins are the most important targets of proposed GBS vaccines [19-21]. The researchers observed that the presence of PI-2a was limited mainly to the MT 18, which significantly associated with serotype III from asymptomatic colonization. In this study, due to the low prevalence of GBS, the number of selected patients were also low. Moreover, in assessing profiling of GBS diversity, it might produce better results if a study is conducted by at least two different methods to accurately monitor epidemic trends at regional levels.

## Conclusion

The examination of 90 isolates from various origins observed significant variations within the distribution of pilus island varieties across phylogenetic lineage and origin, suggesting that the pilus combination has an effect on host specificity and the outcome of the disease. Furthermore, the extensive distribution of PI in various serotypes and MT throughout the GBS population refers to the advancement of the pilus-based GBS vaccines.

## Conflict of interest

None to declare.
